# On the Measurement of Dielectric Losses and Surface Conductivity of Dielectrics in Parallel Plane Test Capacitors

**DOI:** 10.6028/jres.068A.018

**Published:** 1964-04-01

**Authors:** Lothar Frenkel

## Abstract

Thin slabs of dielectric materials are often tested for their dielectric properties in plane parallel plate capacitors. When surface conductivity is present, as for instance in freshly split mica, losses not connected with the bulk of the material arise. The present paper deals with the general theory of such measurements. The system is reduced to an assembly of lumped elements superimposed on distributed transmission lines. The treatment includes the presence of possible airgaps underneath the plates of the test condenser. It is shown that such losses depend on the reciprocal square root of the frequency. Losses due to this effect cannot be eliminated by guard ring measurement and much of the published data on the losses in mica must be reexamined in the light of the present work. Similar considerations may apply in the case of other materials.

## 1. Introduction

The present paper had its origin in an attempt to interpret the results of some recent work on mica described in a paper by S. Ruthberg and L. Frenkel^1^ which will henceforth be referred to as (I). In (I) we describe some results obtained during measurements of the dielectric losses of freshly split mica and attempt to account for these in terms of the physical properties of an assumed surface layer on the mica. The present paper extends and generalizes the theory of loss measurements in the presence of surface conductivity. It is shown that all the features described in (I) may alternatively be accounted for in a simple manner by the surface conductivity of the specimen alone.

The present paper is however a general contribution to the theory of dielectric measurement and the applicability of this theory is not limited to any particular dielectric. Furthermore some loss measurements in mica and other dielectrics should be reexamined in the light of the theory given here to sort out what part of the losses reported may be ascribed to the bulk of the dielectric and what part is in fact due to surface conduction.

Before introducing a specific model for theoretical analysis we shall examine briefly the salient features of the results reported in (I).

**Figure 1:**
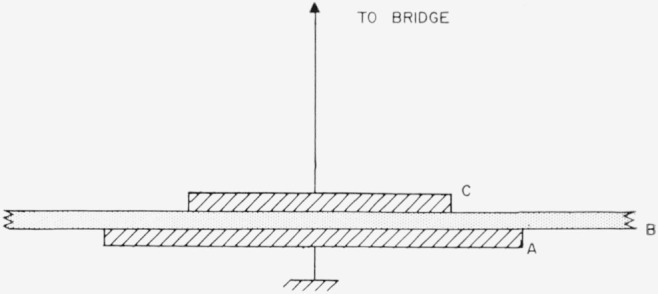
Experimental arrangement for the measurement of dissipation in mica.

The experimental arrangement used is shown in figure 1 and consists of a parallel plate capacitor made up of a large grounded plate (A), a sheet of the dielectric under test (B) and a disk electrode (C) which is connected to a suitable bridge.

Suppose now that a dielectric having one slightly conducting surface is placed in the capacitor with the conducting face resting on the smaller disk (arrangement 1).

It is clear that current will flow away from the edge of the small disk via surface conduction thus giving rise to losses which are added to the losses incurred in the bulk of the material, and it is shown in (I) that these losses are inversely proportional to the square root of the capacitance of the capacitor formed in the manner described above.

The depth of penetration of this edge effect is quite small. This may be seen as follows: A typical value of the surface resistance on freshly split mica^2^ is 10^11^ Ω while the capacitance across a typical sample is 10 *µ*f per cm^2^ thus the resistive component of the impedance along a square of 1 cm edge is in the order of 10^11^ Ω while the shunting impedance across the sample to ground at 1000 c/s is of order 10^7^ Ω. The edge current is therefore quickly attenuated. This fact, as is pointed out in (I) makes it impossible to exclude such losses by means of guardring methods.

Suppose now that the sample is reversed so that the conducting surface is resting on the large, grounded plate (arrangement 2). In this arrangement the field lines all cross the conducting surface at right angles and there are no losses due to surface currents. Since, however, the conductive layer on the surface will also have transverse conductivity one might expect some losses to occur in this case also. The value of the losses in the two arrangements as well as their dependence on the thickness of the dielectric and on the frequency used are subject to physical and instrumental interpretation.

In attempting to explain the losses in the transverse field (arrangement 2) in terms of the physical properties of the surface layer one runs into difficulties. In particular the magnitude of the losses is such that one must assume either a dielectric constant smaller than 1 or a thickness of the layer of the order of millimeters. Neither of these conditions is reasonable.

The salient experimental features which any theory of this effect must explain are listed below.

- The loss induced by splitting is *proportional* to the capacitance of the piece when measured with the fresh side in contact with the large plate (arrangement 2).
- If the fresh side is placed in contact with the smaller plate the losses increase due to the edge effect. This difference in loss is found to be *inversely proportional* to the square root of the capacitance of the piece.
- All induced losses vary as the inverse square root of the frequency.

The order of magnitude of the losses with the two arrangements is the same.

One further feature of the experiments must now be described since it provided the clue for the present solution of the problem. If one examines the capacitance of the test capacitor with progressively thinner pieces of the mica between the plates one finds upon plotting the inverse of the capacitance versus the thickness of the dielectric that the resulting straight line does not go through the origin but has, at zero thickness, a positive intercept. This indicates that in practice there is a fixed effective airgap between the plates of the test capacitor and the dielectric. Causes for the airgap may either be inherent in the mica surface or of an instrumental nature. Whatever its origin however the existence of the airgap suggests that surface currents will occur also when the lossy surface is exposed to the large plate since there will be currents along the surface wherever the surface contact with the plate is interrupted. In (I) the existence of the gap was taken into account by a correction applicable for measurements in uniform airgaps but no actual losses at the contact edges underneath the plates were assumed to occur.

The magnitude of the correction is quite large and corresponds, for thin samples, to airgap thicknesses amounting to several percent of the thickness of the specimen under test. Consequently the losses associated with this type of contact current cannot be neglected.

## 2. Description of the Model

We are now ready to set up a model representative of the arrangement described above. In practice there may be many areas of contact under the plates and their shape and thickness may be quite varied. Remembering however that the attenuation is quite rapid and that the losses in the various gaps may be summed we need to treat only one typical case, i.e., the loss in a cavity under the plates. It will be easily seen that the edge loss is only a special case of the general model, namely the special case where the airgap capacitance has gone to zero.

**Figure 2:**
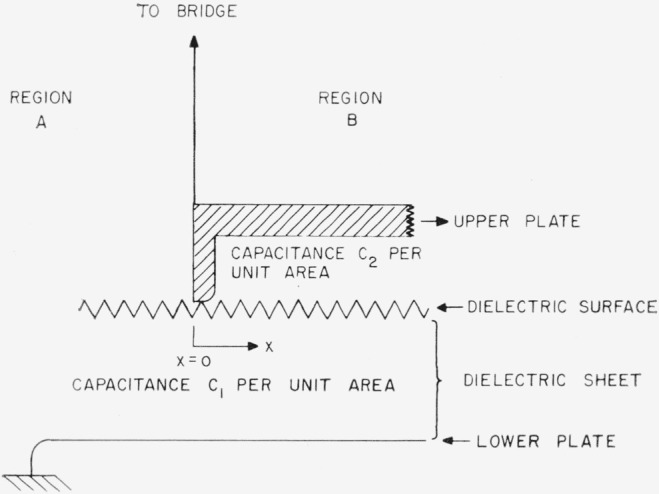
Equivalent circuit representing the mica capacitor near the edge of the upper plate Only the edge of the upper plate touches the mica surface

The equivalent circuit with the airgap under the plates is indicated in figure 2. *A* represents the region beyond the edge of the smaller plate where the capacitance of the sample is in series with the resistance of the surface. The region *B* represents the area under the small plate where the resistive path is shunted by the effective airgap. *C*_1_ is the capacitance across unit area of the sample, *R* is the surface resistivity of a unit square of the surface and *C*_2_ is the capacitance across unit area of the airgap.

Since the edge effects are confined to a small area near each contact edge one may regard the surface near the edge as forming a part of an infinite parallel plane transmission line, of width equal to the length of the respective edge. In the case of the edge of the upper plate this length is the circumference of the plate but under the airgap one cannot determine the length of the contact edges directly. The qualitative features of the model do not depend on the length of these edges however, and the actual lengths required to account for the phenomena described in (I) are of the order of the plate circumference which is entirely reasonable.

Because the attenuation is very high we may assume that the characteristic inductance of the line is negligible and that the progress of the wave into the line is slow. The progress of the signal along the metal plates by contrast is infinitely fast. These assumptions may be made plausible as follows: Assume that a potential *V* is suddenly applied to the upper plate. The potential *V* will be established across the entire upper plate with a speed close to the speed of light and the surface of the mica will assume a potential determined by the capacitance divider consisting of *C*_1_ and *C*_2_. After a time determined by the time, constant (*C*_1_*R*) the surface of the dielectric will however reach the potential *V* by conduction along the surface. Our assumption now implies that this time is long compared to the time required to establish initial equilibrium in the upper plate.

## 3. Theory

In practice a sinewave in a frequency range from 10 to 1000 c/s is used for this experiment and the upper plate is therefore at potential:

$$V\left(t\right)=V_{0}e^{i\;\omega t}.$$ (1)

Our task is to calculate the in-phase component of the current in section *B.*

Our plan of attack is the following. We shall establish transmission line equations for the surface potential *V_s_*(*x*,*t*) and the surface current I*_s_*(*x,t*) in order to find the current into the line, I*_s_*(*o*,*t*). To this we will add the current flowing at any time into the airgap. This current is determined for each point *x* on the surface by the potential difference (*V*(*t*) — *V_s_*(*x,t*).). The airgap current must therefore be obtained as an integral over the entire line. This transmission line is described by:

$$\frac{\partial I_{s}}{\partial x}=-C_{1}\frac{\partial V_{t}}{\partial t}+C_{2}\left(\frac{\partial Vt}{\partial t}-\frac{\partial V_{s}}{\partial t}\right)$$ (2)

and

$$\frac{\partial V_{s}}{\partial x}=-I_{s}R.$$ (3)

The solution can easily be guessed. We note that for large values of *x, V_s_* must approach a fixed value.

$$\underset{x\to \infty }{\lim}V_{s}\left(x,t\right)=V_{0}e^{i\;\omega t}\frac{C_{2}}{C_{1}+C_{2}}$$ (4)

while at *x*=0

$$V_{s}\left(0,t\right)=V_{0}e^{i\;\omega t};$$ (5)

accordingly we try:

$$V_{s}=V_{0}e^{i\;\omega t}\left[e^{-x\left(\alpha +ik\right)}\left\{1-\frac{C_{2}}{C_{1}+C_{2}}\right\}+\frac{C_{2}}{C_{1}+C_{2}}\right]$$ (6)

since this function has the correct limiting dependence. Further we assume

$$I_{s}=I_{s,0}e^{i\;\omega t-x\left(\alpha +ik\right)};$$ (7)

substitution of the trial solutions (6) and (7) into eqs (2) and (3) gives

$$I_{s,0}R=V_{0}\left(\alpha +ik\right)\left[\frac{C_{1}}{C_{1}+C_{2}}\right]$$ (8)

and

$$I_{s,0}\left(\alpha +ik\right)=V_{0}i\omega C_{1};$$ (9)

from (8) and (9) we obtain by elimination of (*α + ik*)

$$I_{s,0}=V_{0}\sqrt{\frac{i\omega C_{1}}{R}}\sqrt{\frac{C_{1}}{C_{1}+C_{2}}}.$$ (10)

The loss factor depends on the real part of this expression.

We must next calculate the real part of the current entering the capacitance *C*_2_ via the metal surface above the airgap. This current is given by:

$$I_{C_{2}}=C_{2}\int_{0}^{z}\frac{\partial }{\partial t}\left(V\left(t\right)-V_{s}\right)dx$$ (11)

where *z* is the extent of the line.

Using our solution (6) for *V_s_* this becomes:

$$I_{C_{2}}=i\omega \frac{C_{1}C_{2}}{C_{1}+C_{2}}V\left(t\right)\int^{z}\left(1-e^{-x\left(\alpha +ik\right)}\right)dx$$ (12)

we note that for very high resistivity the integral is

$$I_{C_{2}}=i\omega V\left(t\right)\frac{C_{1}C_{2}}{C_{1}+C_{2}}z$$ (13)

as expected.

We are however interested in the general case and particularly in the real amplitude

$$I_{C_{2,0}}\;\text{real}=-\frac{C_{1}C_{2}}{C_{1}+C_{2}}V_{0}\omega \int_{0}^{z}\sin\;kxe^{-\alpha x}dx.$$ (14)

Now *α* may be found from (8) and (9) to be

$$\alpha =k=\frac{1}{\sqrt{2}}\sqrt{\omega R\left(C_{1}+C_{2}\right)}$$ (15)

and the integral (14) evaluated for a sufficiently large upper limit gives

$$I_{C_{2,0}}\;\text{real}=-\frac{\omega V_{0}}{2\alpha }\frac{C_{1}C_{2}}{C_{1}+C_{2}}$$ (16)

combining (15) and (16) gives

$$I_{C_{2,0}}\;\text{real}=-\sqrt{\frac{\omega C_{1}}{2R}}\sqrt{\frac{C_{1}}{C_{1}+C_{2}}}\left(\frac{C_{2}}{C_{1}+C_{2}}\right).$$ (17)

The total “in phase current” per unit edge of section *B* consists of the real parts of eqs (17) and (10), i.e.,

$$I\;{\text{real}}_{B}=\sqrt{\frac{\omega C_{1}}{2R}}{\left(\frac{C_{1}}{C_{1}+C_{2}}\right)}^{3/2}.$$ (18)

In section *A, C*_1_ is unchanged and *C*_2_ = 0 so that from (18)

$$I\;{\text{real}}_{A}=\sqrt{\frac{\omega C_{1}}{2R}}.$$ (19)

This formula was reported in (1) but may now be regarded as a special case of (18).

We must next determine the total current in the capacitor so as to form the loss factor. This current will arise from contributions of many sections of the capacitor having various values of *C*_2_ but since we can only measure an average value of *C*_2_ we proceed as if *C*_2_ were the same at all points, then

$$I\;\text{imag}=\omega V_{0}\left(\frac{aC_{1}C_{2}}{C_{1}+C_{2}}\right)$$ (20)

where *a* is the area of the capacitor, i.e., the area of the small plate.

We are now in a position to calculate separately the loss tangents due to sections *A* and *B* of figure 2. Dividing (18) and (19) by (20) and multiplying each of the resulting expressions by an effective edge length gives:

$$\phi_{B}=\frac{l_{B}}{a}\frac{C_{1}}{C_{2}}\sqrt{\frac{1}{2R\omega }}\sqrt{\frac{1}{C_{1}+C_{2}}}$$ (21)

and

$$\phi_{A}=\frac{l_{A}}{a}\frac{1}{\sqrt{C_{1}}}\sqrt{\frac{1}{2R\omega }}\left(\frac{C_{1}+C_{2}}{C_{2}}\right).$$ (22)

## 4. Discussion

For large values of *C*_2_ eqs (21) and (22) reduce to

$$\phi_{B}\propto C_{1}1/\sqrt{\omega }$$ (23)

and

$$\phi_{A}\propto \left(1/\sqrt{C_{1}}\right)\left(1/\sqrt{\omega }\right)$$ (24)

respectively. If now the sample has its conductive face on the larger plate, there are no edge effects and (23) is applicable thus confirming that much of the experimental observations. If on the other hand, the sample is reversed, then both effects operate and the difference will be due to *ϕ_A_* thus confirming the remainder of the observed dependence on capacitance.

In practice the approximations (23) and (24) are not justified since *C*_1_ and *C*_2_ are generally of the same order, but it must be remembered that the correction as used in (I) is now included in (21) and (22). A check on the results in (I) showed that agreement with experiment is just as good using (21) and (22) on the experimental values that were quoted in (I) provided that the corrections used there are discarded.

**Figure 3:**
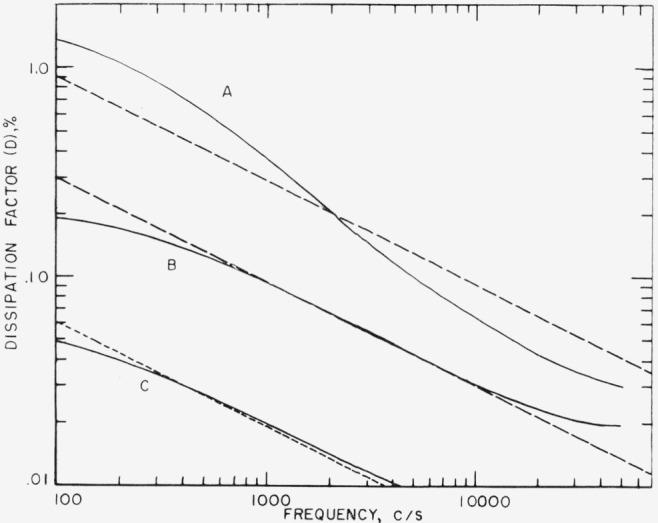
Relationship between the dissipation in a mica capacitor and frequency for various experimental arrangements.

It also follows from eqs (21) and (22) that the losses due to tangential current in the surface all depend on the inverse of the square root of the frequency. We reproduce in figure 3 the frequency dependence of the losses of split and unsplit mica together with arbitrarily drawn lines of slope (−1/2). The agreement of the two curves representing split samples is very gratifying. Of considerable interest is the fact that the curve for the old surface also follows the −1/1 power law. This indicates that all losses measured in mica are possibly due to surface currents and the intrinsic losses in this material may be much smaller than values usually quoted in the literature.

The final question arises whether the effective airgap is physical or instrumental. If it were physical we would have to suppose that the mica surface is uneven to a sufficient degree to cause the effective airgap. If the explanation were instrumental one would have to suppose that the mica sample, though smooth, did not make good contact with the plates perhaps because of a warping in the plates. In any case it is hoped that the theory presented here will be generally useful in the measurement of dielectric properties of thin samples.

## 5. Conclusions

The present work indicates that whenever thin slabs of material are examined for loss characteristics, one must expect disturbing influences due to surface losses. These generally depend on the inverse square root of the frequency. Though the theory is developed specifically to account for a rather esoteric experiment with mica, its general significance for dielectric measurements is obvious.

## Footnotes

1 S. Ruthberg and L. Frenkel, Dielectric behavior of the film formed on mica cleaved in moist air, J. Res. NBS 68A (Phys. and Chem.) No. 2, 173 (1964).

2 J. M. MaCaulay and D. Carson, J. Roy. Tech. Coll., Glasgow 2, 161 (1930).

